# p53 Isoforms in Cellular Senescence- and Ageing-Associated Biological and Physiological Functions

**DOI:** 10.3390/ijms20236023

**Published:** 2019-11-29

**Authors:** Kaori Fujita

**Affiliations:** Cell Induction and Regulation Field, Department of Clinical Application, Center for iPS Cell Research and Application, Kyoto University, 53 Kawahara-cho, Shogoin, Sakyo-ku, Kyoto 606-8507, Japan; kaori.fujita@cira.kyoto-u.ac.jp; Tel.: +81-75-366-7087

**Keywords:** p53 isoform, cellular senescence, ageing and age-related diseases, reprogramming, cancer

## Abstract

Cellular senescence, a term originally used to define the characteristics of normal human fibroblasts that reached their replicative limit, is an important factor for ageing, age-related diseases including cancer, and cell reprogramming. These outcomes are mediated by senescence-associated changes in gene expressions, which sometimes lead to the secretion of pro-inflammatory factors, or senescence-associated secretory phenotype (SASP) that contribute to paradoxical pro-tumorigenic effects. p53 functions as a transcription factor in cell-autonomous responses such as cell-cycle control, DNA repair, apoptosis, and cellular senescence, and also non-cell-autonomous responses to DNA damage by mediating the SASP function of immune system activation. The human *TP53* gene encodes twelve protein isoforms, which provides an explanation for the pleiotropic p53 function on cellular senescence. Recent reports suggest that some short isoforms of p53 may modulate gene expressions in a full-length p53-dependent and -independent manner, in other words, some p53 isoforms cooperate with full-length p53, whereas others operate independently. This review summarizes our current knowledge about the biological activities and functions of p53 isoforms, especially Δ40p53, Δ133p53α, and p53β, on cellular senescence, ageing, age-related disorder, reprogramming, and cancer. Numerous cellular and animal model studies indicate that an unbalance in p53 isoform expression in specific cell types causes age-related disorders such as cancer, premature ageing, and degenerative diseases.

## 1. Introduction

**Figure 1:**
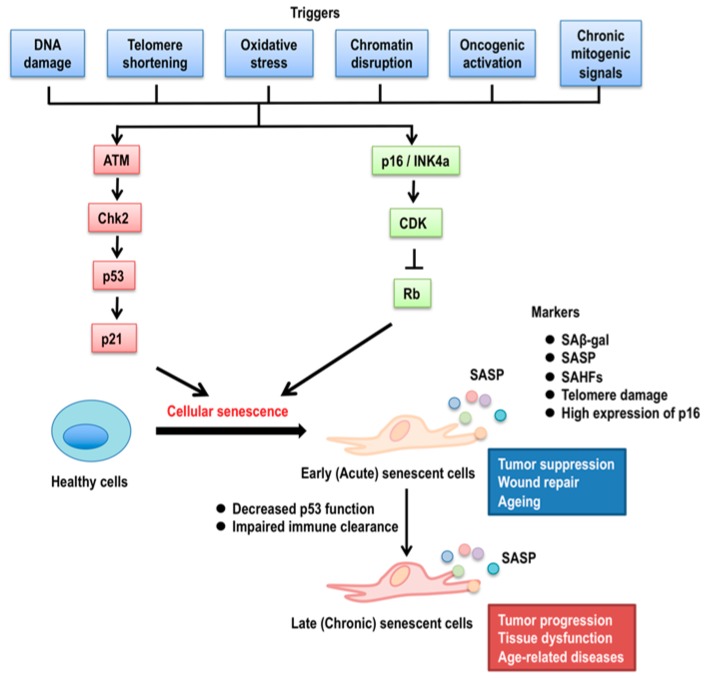
Mechanisms of cellular senescence. The many triggers for cellular senescence, such as DNA damage, telomere shortening, oxidase stress, chromatin disruption, and oncogenic activation, can initiate p53 signaling pathways through the activation of ATM (ataxia telangiectasia-mutated) kinase and ATM-mediated Chk2 (check point kinase 2). Activated Chk2 phosphorylates p53, which protects p53 from Mdm2 (mouse double minute 2)-mediated protein degradation. Oncogenic activation and chronic mitogenic signals induce p16^INK4a^ activation, resulting in the inhibition of CDK (cyclin-dependent kinase) activity. Increased p21^Waf1/Cip1^ expression and/or Rb (retinoblastoma) activity cause cellular senescence. Senescence markers include senescence-associated β galactosidase activity (SAβ-gal), senescence-associated secretory phenotype (SASP), senescence-associated heterochromatic foci (SAHFs), telomere dysfunction, and the high expression of p16^INK4a^. Early (acute) senescent cells self-organize their elimination by the immune system through SASP, which contributes to tumor suppression, wound repair, and probably healthy normal ageing. Late (chronic) senescent cells can evolve from early senescence if the clearance of early senescent cells by the immune system is impaired with age, leading to alterations of SASP, resulting in tumor progression, tissue dysfunction, and aged-related diseases.

Over five decades ago, Hayflick and Moorhead discovered and described the process of cellular senescence in normal human fibroblasts as a limited number of cell divisions, followed by irreversible growth arrest after serial cultivation in vitro [1,2]. Since then, several types of cellular senescence have been identified. Replicative cellular senescence describes a senescent state with telomere shortening or dysfunctional telomeres [3,4], and stress-induced cellular senescence is induced by cellular stresses, such as mitogenic and oncogenic stimuli, namely p38 MAPK activation and overexpression of oncogenic Ras [5,6]. Senescent cells differ from other non-dividing cells (quiescent or terminally differentiated cells) by several markers, such as the expression of p16^INK4A^ [7,8] and senescence-associated β-galactosidase (SA-β-gal) [7,9], senescence-associated heterochromatic foci (SAHFs) [10], which contribute to silencing E2F target genes such as *PCNA* and *cyclin A*, and the senescence-associated secretory phenotype (SASP) [11,12,13], which consists of secreted inflammatory cytokines and other signaling molecules including interleukin-1 (IL-1), IL-6, IL-8, vascular endothelial growth factors (VEGF) [14] and matrix metalloproteinases (MMPs) [15,16]. In general, cellular senescence constitutes a critical mechanism for tumour suppression in vivo and may contribute to organismal aging and age-related diseases. Further, accumulating evidence indicates that the physiological relevance of cellular senescence extends beyond tumor suppression to include several biological processes such as embryonic development [17,18], tissue repair [19,20], and wound healing [20]. Moreover and counterintuitively, recent data strongly suggest that SASP can contribute to not only tumor suppression but also tumor promotion [4,21,22]. The accumulation of senescent cells does not directly determine the organismal lifespan, but it does accelerate with ageing [23,24,25,26]. The increase of senescent cells in aged tissues is thought to cause a functional decline in homeostasis and integrity and is linked with diminished responses to physiological conditions under stress (Figure 1).

p53 is a transcriptional factor highly regulated by post-transcriptional modifications [27,28,29,30]. It regulates cellular senescence, which is important for tumor suppression in vivo and organismal ageing. p53 regulates self-renewal, genome stability, and the differentiation of normal and cancer stem cells. In addition, p53 and retinoblastoma (Rb)-p16^INK4a^ pathways modulate the efficiency of cell reprogramming to induce pluripotent stem cell (iPSC) generation by cellular senescence [31]. p53 knockdown and a p53 dominant-negative mutant were shown to enhance cell reprogramming, while upregulated p53 reduced the cell reprogramming efficiency, showing that p53 activity is critical in reprogramming [32,33,34]. However, p53 is also critical in DNA damage repair, thus its inactivation could result in persistent DNA damage and chromosome aberrations [35,36,37].

p53 directly binds as a tetramer to the p53-response elements on the DNA of more than 3600 estimated target genes [38]. This binding stimulates tumor suppression mechanisms by halting cell proliferation and inducing apoptosis in response to various stresses. Conversely, in unstressed conditions, p53 protein expression is kept low due to E3-ubiquitn ligase Mdm2 (murine double minute 2)-mediated proteasomal degradation [39]. Mdm2 is also directly induced by p53, resulting in a negative feedback loop in p53 signaling. The tight regulation between p53 and Mdm2 is important, because excess p53 can induce cell death in normal cells, whereas insufficient p53 can transform normal cells. Drugs targeting wild-type p53 serve to enhance the stabilization of p53 via several mechanisms: 1) Nutlin 3a, benzodiazepinediones, and spiro-oxindoles target the p53-Mdm2 interaction to reduce Mdm2-mediated proteasomal degradation; 2) RITA (Reactivation of p53 and induction of tumor cell apoptosis) directly binds to p53, inducing a conformational change that inhibits Mdm2 binding; and 3) Mdmx inhibitors, which block Mdmx-Mdm2 dimerization to activate p53 [40]. These drugs induce apoptosis by upregulating several pro-apoptotic p53 target genes, such as PUMA (p53 upregulated modulator of apoptosis), NOXA (Laten for damage), BAX (Bcl-2-associated X protein), and BAK (BCL2-antagonist/killer 1), which are all critical for tumor suppression [41]. Indeed, some of these drugs have been used successfully as chemotherapies, with many inducing p53-mediated apoptosis in tumors. [29,42,43,44].

p53-mediated DNA damage responses (DDR) are also a trigger of cellular senescence and caused by multiple inducers, including not only telomere shortening but also reactive oxygen species (ROS) [30,45], ultraviolet light (UV) [46,47,48], and along with cancer therapies [49]. DDR activate ataxia teleangectasia-mutated (ATM) kinase, which phosphorylates p53 in a checkpoint kinase (Chk) 2-dependent manner, thus accumulating p53 protein due to the avoidance of Mdm2-mediated proteasomal degradation and initiating the transcription of multiple p53 target genes [50]. The first identified senescence-associated downstream target gene of p53 is *CDKN1A* gene, which codes for the cyclin-dependent kinase (CDK) inhibitor p21^Waf1/Cip1^ [51,52,53,54]. p21^Waf1/Cip1^ is an essential mediator of p53-dependent cell cycle arrest following DNA damage [55] (Figure 1). Mouse embryonic fibroblasts lacking p21^Waf1/Cip1^ fail to undergo p53-dependent G1 arrest after DNA damage [55]. Subsequent studies have shown that p53 binds and transactivates the *p21^Waf1/Cip1^* promoter during the replicative cellular senescence of normal human diploid fibroblasts [56]. In fact, the lack of p21^Waf1/Cip1^ prevents cellular senescence in several settings [52,57,58,59]. On the other hand, forced p21^Waf1/Cip1^ expression induces senescence in vitro [60,61]. These studies define p21^Waf1/Cip1^ as a strong mediator of p53-regulated growth arrest and cellular senescence in response to various stresses and DNA damage. 

**Figure 2:**
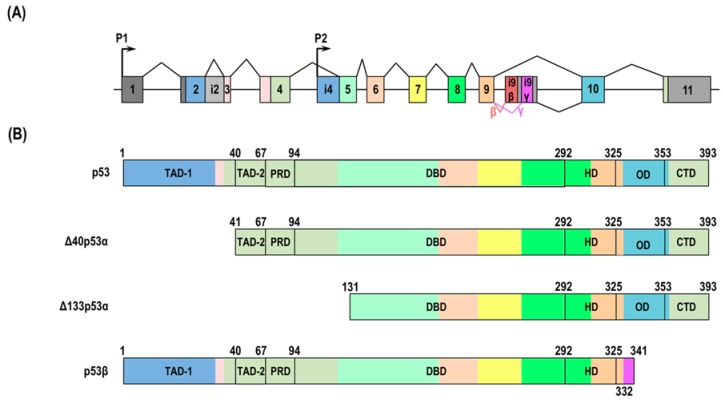
The human *TP53* gene and cellular senescence-associated isoform proteins. (**A**) The human *TP53* gene structure. Boxes indicate exons, and lines indicate introns. The exons and introns are not to scale. Grey boxes show non-coding sequences. Other colors show coding sequences. The human *TP53* gene is composed of 11 exons and encodes several *p53* isoforms using alternative promoters (P1 and P2) and splicing sites (zigzag lines). The gene also includes two unique exons that are part of intron 9 and encode the β and γ isoforms. (**B**) The cellular senescence-associated human p53 isoforms. The colors of the protein domain match the corresponding exons. p53 has two transactivation domains (TAD-1 aa 1–40 and TAD-2 aa 41–67), a proline-rich domain (PRD, aa 68–98), DNA-binding domain (DBD, aa 94–292), oligomerization domain (OD, aa326–353), and carboxy-terminal regulatory domain (CTD, aa 353–393). Δ40p53 lacks TAD1 because of alternative initiation at ATG40. Δ133p53α is transcribed from P2 and lacks the whole N-terminus (TAD-1, TAD-2, and PRD) and part of DBD. p53β is missing several residues that are replaced by new amino acids through the alternative splicing of intron 9.

p53 isoforms were first discovered by Matlashewski in 1984 [62]. Wolf et al. showed alternatively spliced C-terminal variants of mouse p53 in 1985, and their results were confirmed in several human cells [63,64,65]. The human full-length p53 protein is composed of 393 amino acids with six classified domains: transcription activation domain (TAD) I (residues 1–40) and TAD II (residues 41–67), which interact with various proteins; a proline-rich domain (residues 68–98), which is conserved in most p53 isoforms; DNA-binding domain (DBD) (residues 94–292); hinge domain (HD) (residues 293–325); oligomerization domain (OD) (residues 326–353); and carboxy-terminal regulatory domain (CTD) (residues 353–393) [66,67,68,69] (Figure 2A). Bourdon et al. recognized that the human *TP53* gene structure is similar to human *TP63* and *TP73* genes and discovered that human *TP53* gene encodes at least twelve natural isoforms including the full-length p53 protein due to alternative initiations of translation, usage of alternative promoters, and alternative splicing [70] (Figure 2B). *p53* mRNA isoforms are expressed in a tissue-specific manner. For example, while *Δ133p53α* is expressed in most normal tissues except the prostate, uterus, skeletal muscle, and breast, *p53β* is expressed in most normal tissues but the brain, lung, prostate, skeletal muscle, spinal cord, and fetal liver. 

The biological activities of p53 isoforms differ. p53β preferentially binds to p53-responsive elements in the promoters of *p21^Waf1/Cip1^* and *Bax* but not of *Mdm2*, whereas full-length p53 preferentially binds to p53-responsive elements in the promoters of *Mdm2* and *p21^Waf1/Cip1^* but not of *Bax in vitro*. Under stress conditions, p53β complexes with full-length p53 to enhance the transcriptional activity of full-length *p53* against *Bax* promoter, suggesting that p53β cooperates with full-length p53 [70]. Another in vitro experiment showed that the co-transfection of Δ133p53α with full-length p53 strongly inhibits p53-mediated apoptosis in a dose-dependent manner, indicating that Δ133p53α has an inhibitory regulation on full-length p53 [70,71]. Because p53 isoforms have tissue-specific expression and activity that are tightly and differentially regulated, the balance of their expression and function makes p53 isoforms critical for p53-mediated cellular or tissue outcomes. This review focuses on the contribution of p53 isoforms to cellular senescence, ageing, cancer, and cell reprogramming, by examining how the isoforms interact with full-length p53.

## 2. p53 Isoforms in Cellular Senescence

**Figure 3:**
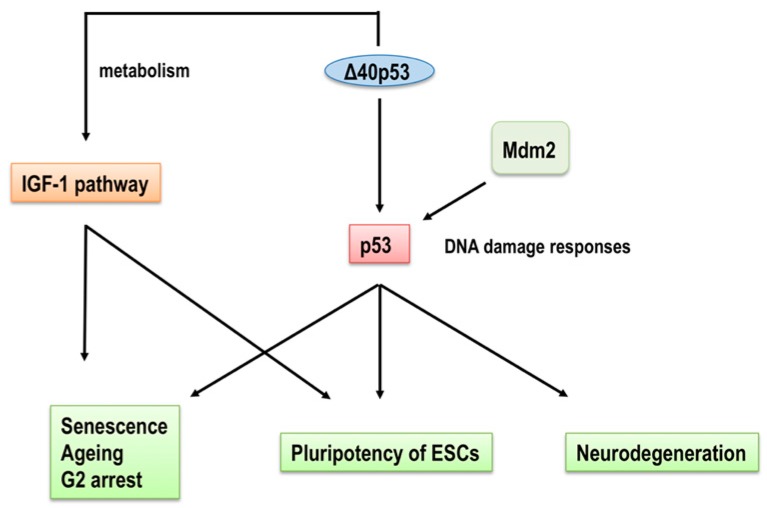
A model for the regulation of cellular senescence and ageing by Δ40p53. Δ40p53 directly regulates the IGF-1 signaling pathway to modulate cell growth and survival factors. On the other hand, the binding of Δ40p53 to full-length p53 regulates the transcriptional activity of full-length p53 on target genes and its capacity to bind Mdm2 for proteasomal degradation. Regulation of the IGF-1 signaling pathway and full-length p53 by Δ40p53 affects not only cellular senescence and ageing but also the pluripotency of ESCs and neurodegeneration.

Δ40p53 (also known as ΔNp53 or p47) was the first described human p53 isoform and is derived from the alternative translation initiation of *p53* mRNA at the second AUG codon [70,72,73,74]. This isoform does not contain the Mdm2-binding site or N-terminal transactivation domain of full-length p53. Mdm2 induces the translation initiation of full-length *p53* and *Δ40p53*, however, it also degrades full-length p53, while Δ40p53 stabilizes full-length p53 in the presence of Mdm2 [72]. Candeias et al. later showed that full-length p53 and Δ40p53 were separately and competingly regulated, so that *Δ40p53* was normally masked by cap-dependent translation initiation [75,76]. Endoplasmic reticulum stress induces *Δ40p53* mRNA translation and its homo-oligomerization to induce G2 cell cycle arrest. In contrast, full-length p53 induces G1 arrest [77,78]. In relation to senescence, the proliferation of embryonic cells in mice expressing transgenic p44 (a mouse homolog of Δ40p53) was decreased by the induction of p21^Waf1/Cip1^ compared with embryonic cells in wild-type and heterozygous mice [79]. Mouse embryonic fibroblasts (MEF) from p44 transgenic mice experiencing oxidative stress, which is an inducer of cellular senescence, by treatment with H_2_O_2_ showed less cell proliferation and were more SA-β-gal-positive, indicating that the overexpression of p44 induced cell cycle arrest and cellular senescence [80]. Furthermore, neuronal stem/progenitor cells in the p44 transgenic mice showed reduced cell proliferation without increased apoptosis, suggesting that defects in cell proliferation limit stem cell self-renewal and cause premature stem cell depletion [81]. In contrast to somatic stem cells, cell growth rates under the ectopic expression of p44 (p44Tg) in embryonic stem cells (ESCs) were similar with normal ESCs, but the loss of one copy of p44 in ESCs significantly decreased cell proliferation and pluripotency. The Δ40p53 expression level controls the switch from pluripotent ESCs to somatic cells by regulating the activity of full-length p53 at target genes (Nanog and IGF-1 (Insulin like growth factor 1) receptor) [82]. Furthermore, along with in normal cells, the exogenous expression of both Δ40p53 and wild-type p53 in human hepatocellular carcinoma cell lines reduced cell growth and induced senescence by increasing the expression of p21^Waf1/Cip1^ and IL-8 to stabilize full-length p53 [83] (Figure 3).

**Figure 4:**
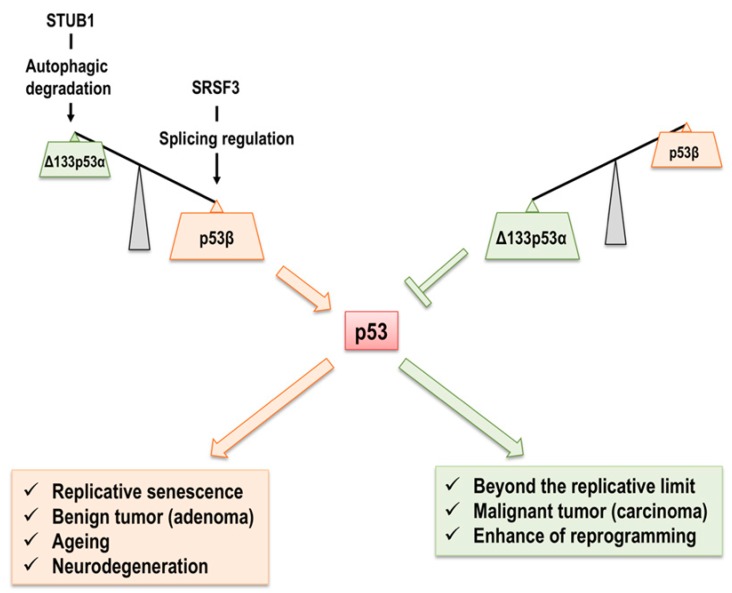
A model for the regulation of cellular senescence, ageing, and age-related disorders by Δ133p53α and p53β. Abundant Δ133p53α competitively acts on p53 functions in proliferating cells, and p53β expression is kept at low levels. In senescent cells, *p53β* is upregulated by SRSF3-mediated splicing, and Δ133p53α is downregulated by STUB1-mediated chaperon-dependent autophagic degradation. Change in the senescence-associated p53 isoform expression also contributes to tumor progression from adenoma to carcinoma along with neurodegeneration and reprogramming into iPSCs.

The isoform that is most associated with cellular senescence is Δ133p53α. Δ133p53α is derived from the internal initiation of transcription at the intragenic promoter located at intron 4, resulting in specific mRNA. The first AUG that is used for the initiation of translation corresponds to codon 133 of full-length *p53*. Δ133p53α lacks the first 132 amino acids, TAD I, TAD II, as well as the first 30 residues of DBD [70]. We have shown that Δ133p53α is abundant in early passage normal human fibroblasts and decreases in late passage and senescent cells. Interestingly, siRNA (short interfering RNA)-mediated knockdown of endogenous Δ133p53α induces cellular senescence, which is attributed to the induction of *p21^Waf1/Cip1^* and other p53 transcriptional target genes, including *microRNA-34a*. In contrast, the overexpression of Δ133p53α in late passage (near senescent) normal human fibroblasts extends the cellular replicative lifespan due to the inhibited expression of *p21^Waf1/Cip1^* and other p53 transcriptional target genes [84]. However, premature senescence induced by oncogenic Ras or acute telomere dysfunction is not associated with diminished Δ133p53α [84]. The downregulation of Δ133p53α in replicative senescence is not because of a change in mRNA levels or proteasomal degradation. Instead, unlike full-length p53, which is degraded by the Mdm2-mediated proteasomal pathway, Δ133p53α is degraded by autophagy [85,86]. The chaperone-associated E3 ubiquitin ligase STUB1 (STIP1 homology and U-box containing protein 1), which is known to regulate autophagy, interacts with Δ133p53α and is downregulated in replicative senescence. Thus, in early passage human normal fibroblasts, Δ133p53α interacts with STUB1 to inhibit the recruitment of Δ133p53α to the autophagosome. In contrast, the dysregulation of STUB1 in senescent cells can release Δ133p53α from the STUB1 complex and recruit it to the autophagosome, resulting in the degradation of Δ133p53α [86]. Along with replicative senescent human normal fibroblasts, radiation-induced senescent astrocytes show decreased Δ133p53α levels. The overexpression of Δ133p53α in human astrocytes protects radiation-induced cellular senescence, resulting in the inhibition of astrocyte-mediated neuroinflammation via the promotion of DNA repair [87]. Δ133p53α in a human hepatocyte cell line (QSG-7701) is induced by γ-irradiation, but not other stresses such as heat shock or UV irradiation, to promote DNA double-strand break repair, where Δ133p53α upregulates the transcription of the repair genes *RAD51*, *LIG4*, and *RAD52* by binding to a p53-responsive element in their promoters. QSG-7701 cells with Δ133p53α-knockdown eventually arrest at the G2 phase in response to γ-irradiation and ultimately become senescent [88]. Δ133p53α is transactivated by p53, p63, and p73 isoforms after genotoxic stress [89]. In addition, Δ133p53α has been shown to regulate gene expression in both a full-length p53-dependent and -independent manner [90] (Figure 4).

p53β, which is obtained from the P1 promoter of *TP53* gene and alternative splicing of intron 9, is upregulated in normal human senescent fibroblasts [70,84]. It was also found that the overexpression of p53β induced cellular senescence in early passage by the upregulation of p53 target genes such as *p21^Waf1/Cip1^* via cooperation with full-length p53 [84]. The downregulation of SRSF3 (serine and arginine rich splicing factor 3, SRp20), which is a member of a highly conserved family of splicing factors and sequence-specifically binds to the *p53β*-unique exon i9β on *p53* pre-mRNA to prevent the induction of p53β in proliferating normal human fibroblasts (Figure 2), induces p53β at the mRNA and protein levels, because SRSF3 can leave an alternative exon in *p53β* mRNA during replicative senescence. Indeed, knockdown of SRSF3 in early-passage normal human fibroblasts induces senescence, which is partially rescued by full-length *p53*, suggesting that SRSF3 acts on p53-mediated cellular senescence [91]. I propose that the balance between endogenous p53β and Δ133p53α in normal human fibroblasts is critical for the regulation of replicative cellular senescence. Finally, the ectopic expression of p53β in RKO and MCF-7 cancer cell lines is unable to modulate p53-dependent stress responses including infrared radiation (IR)-induced senescence [92]. Further studies are needed to clarify the p53β-mediated mechanism for senescence induction, including the cell type affected by p53β and the manner with which p53β induces senescence under different stresses (full-length p53-dependent or -independent) (Figure 4).

## 3. p53 Isoforms in Ageing and Age-Related Functional Decline

Transgenic mice overexpressing Δ40p53 show small body size and ageing phenotype, including typical lordokyphosis, and reduced bone density. However, these effects are not seen with the same transgenic mice in p53 null background, suggesting that Δ40p53 is dependent on the presence of full-length p53 [79]. Moreover, the phenotype of Δ40p53 transgenic mice alters insulin-like growth factor (IGF) signaling, which is associated with the regulation of ageing [93,94,95,96]. Serum IGF levels were elevated in Δ40p53 transgenic mice more than three-months-old but not in younger mice, and IGF-1 receptor expression levels and activated Akt levels, a downstream target of IGF1, were also upregulated in older Δ40p53 transgenic mice, suggesting that the IGF signaling pathway is altered with an increase in Δ40p53 levels. Additionally, the upregulated IGF signaling pathway in Δ40p53 transgenic mice led to the phosphorylation of p53 at Ser15, resulting in the enhanced the stabilization and transcriptional activity of p53 to induce *p21^Waf1/Cip1^* and *Mdm2* through sustained ERK (extracellular signal-regulated kinase) activation [79]. It also led to cell cycle arrest via the activation of ERK signaling, which in turn inhibited cell proliferation. Therefore, the small size of Δ40p53 transgenic mice was caused by decreased cell number, which consequently caused cellular senescence and premature ageing phenotypes [79]. New neurons in the olfactory bulb of the older Δ40p53 mice were reduced compared to wild-type due to the accelerated decline of proliferating cells and stem cells in the subventricular zone by the constitutive activation of full-length p53 and subsequent constitutive expression of p21^Waf1/Cip1^ in neural stem cells [81]. Mice 2.5-months old and homozygous for a transgene encoding Δ40p53 showed memory and synaptic defects because of IGF-1 receptor hyperactivation and abnormal tau metabolism [97]. The expression of a humanized form of mouse amyloid precursor protein (hAPP) in Δ40p53 transgenic mice also reduced lifespan and degenerated memory-forming and -retrieving areas of the brain compared to hAPP-expressing wild-type mice [97]. Thus, the role of Δ40p53 in ageing is two parts. One has Δ40p53 as a regulator of full-length p53 function by complexing with it, resulting in the capacity to transactivate target genes and to bind Mdm2 to undergo proteasomal degradation. The other has Δ40p53 directly regulating the IGF-1 signaling pathway, mediating cell growth and survival in many tissues (Figure 3).

Isolating and manipulating senescent cells from human solid tissues are difficult, complicating study of the in vivo roles of senescent cells in physiological and pathological ageing phenotypes in humans. In contrast, late-differentiated CD8^+^ T lymphocytes from healthy human donors are more easily isolated and manipulated. In addition, late-differentiated CD8^+^ T lymphocytes are observed to accumulate age-dependently and associated with specific changes in cell surface antigen expressions (i.e., the loss of CD28 and gain of CD57) [98,99,100,101,102] as well as other senescence markers, such as SA-β-gal activity, shortened telomeres, increased SAHFs, and increased SASP. In addition, we observed that the in vivo accumulation of senescent CD8^+^ T lymphocytes (CD28^−^CD57^+^), which show the senescence-associated p53 isoform expression signature (diminished Δ133p53α levels and induced p53β levels) in blood during physiological ageing [103]. Cultured CD8^+^ T lymphocytes underwent replicative senescence that was associated with the loss of CD28 and Δ133p53α, which was rescued by the ectopic expression of CD28 or Δ133p53α, respectively, resulting in restored cell proliferation, extended replicative lifespan, and reduced senescent phenotypes. In contrast, Δ133p53α knockdown or p53β overexpression in CD8^+^CD28^+^ cells reduced cell proliferation and induced senescence [103]. This study indicates a role for Δ133p53α and p53β in the regulation of cellular proliferation and senescence that is associated with physiological ageing in vivo (Figure 4).

The senescence-associated p53 isoform expression signature correlates with several age-related disease. The onset of neurodegenerative diseases, such as Alzheimer’s diseases (AD) and sporadic amyotrophic lateral sclerosis (ALS), is associated with ageing and caused by the dysfunction of cross-talk between astrocytes and neurons [104,105]. Astrocytes are the most abundant cell type in the brain and have roles in providing functional and metabolic support to neurons [106]. During the replicative senescence of primary human astrocytes, the senescence-associated p53 isoform signature along with autophagic degradation and the SRSF3-mediated regulation of p53β were observed. These same phenotypes were also observed in the replicative senescence of normal human fibroblasts [87]. Interestingly, neurons co-cultured with Δ133p53α-knockdown or p53β-overexpressing astrocytes showed increased cell death, whereas neurons co-cultured with aged Δ133p53α-overexpressing astrocytes were protected from senescence and cell death. This study also showed that brain tissues from AD and ALS patients had increased numbers of senescent astrocytes that showed less Δ133p53α and more p53β expression, demonstrating in vitro observations are consistent with the in vivo pathology of these neurodegenerative diseases, which has implications in the development of therapeutic interventions [87] (Figure 4).

The premature ageing disorder Huntchinson–Gliford Progeria Syndrome (HGPS) is an extremely rare genetic disorder caused by a *de novo* point mutation in exon 11 of the *LMNA* gene, leading to the increased expression of a truncated splicing mutant of lamin A protein named progerin [107,108]. The accumulation of progerin induces cellular senescence associated with increased DNA damage signaling [109,110,111,112]. Particularly, DNA damage in HGPS is induced by the accumulation of unrepaired DNA double-strand breaks due to defective DNA repair and genomic instability by progerin [113,114]. Near-senescent HGPS fibroblasts express low levels of Δ133p53α and high levels of p53β, while the overexpression of Δ133p53α in near-senescent HGPS fibroblasts delays replicative senescence despite progerin expression levels and nuclear abnormalities remaining unchanged [115]. Δ133p53α promotes the repair of DNA double-strand breaks due to the increased expression and recruitment of RAD51, which is a DNA repair factor essential for effective homologous recombination, through the repression of full-length p53 and upregulation of E2F1, a transcription activator of *RAD51*. Therefore, the restoration of Δ133p53α expression may be a novel therapeutic strategy for treating ageing-associated phenotypes of HGPS in vivo [115] (Figure 4).

## 4. p53 Isoforms in Cell Reprogramming to Pluripotent Cells

Pluripotency and differentiation potential are crucial for cell and tissue homeostasis and regeneration. p53 regulates pluripotency and differentiation through the transcriptional regulation of its target genes [55,116]. Indeed, several studies showed that reducing p53 activity increased the reprogramming efficiency of various mouse and human somatic cells and the self-renewing potential of iPSCs and ESCs [56,57,58,60,117]. These results are attributed to the functions of p53 and to cellular senescence acting as a barrier to cell reprogramming in vitro in a cell-autonomous manner. On the other hand, p53 is also a critical regulator of DNA damage response and repair. These properties have a bigger effect on iPSCs and ESCs than somatic cells because iPSCs and ESCs give rise to various lineage-committed somatic stem/progenitor cells [59,61,118]. To maintain genomic stability, iPSCs and ESCs have high rates of apoptosis to eliminate damaged cells, a function that is also regulated by p53 [119,120]. The expression of Δ133p53α protein in 20 human iPSC and ESC lines is higher than in human normal fibroblasts derived from the iPSC lines, in spite of the widely varied expression levels of full-length p53 among lines [121]. During the process of reprogramming, Δ133p53α protein and its transcript were induced from nine days after the transduction of the Yamanaka factors (Oct4, Klf4, c-Myc, and Sox2) [122]. The overexpression of Δ133p53α enhanced the reprogramming of normal human fibroblasts to iPSCs due to the inhibition of p53-inducible genes that mediate factors for cellular senescence, such as *p21^Waf1/Cip1^*, *PAI-1* (plasminogen activator inhibitor-1), *IGFBP7* (insulin-like growth factor binding protein 7), and *microRNA-34a* [121], and also genes mediating DNA double-strand break repair, such as *RAD51*, *RAD52*, and *LIGASE4* [122]. Karyotype assay [122] and whole-exome sequencing [121] revealed that the overexpression of Δ133p53α led to fewer chromosomal aberrations and somatic mutations than full-length p53 knockdown. These studies demonstrated that the overexpression of Δ133p53α is non- or less oncogenic and mutagenic than the total inhibition of p53 due to the selected induction of p53-mediated genes.

## 5. p53 Isoforms in Cancer

Mice with the loss of a single copy of *Trp53* or *p16 ^INK4a^* are prone to tumors [123,124], but mice carrying an extra copy of either gene are cancer resistant [125,126]. Most, if not all, cancers harbor mutations in one or both pathways in humans [127,128]. Accordingly, these two pathways are crucial anticancer mechanisms that prevent the growth of neoplastic transformed cells, and cellular senescence depends on both [129,130,131]. Cellular senescence also contributes to arresting tumors at the premalignant stage. Senescent cells are detectable in benign tumors, which depending on the tissue type are also known as adenomas and intraepithelial neoplasias [132]. The acute activation of p53 in hepatocellular carcinomas and sarcomas induces senescence, which is followed by tumor elimination [133,134]. Yet cellular senescence paradoxically has a function for tumor promotion, which is probably related to SASP factors. Senescent cells secrete SASP factors, which have been described to reinforce the senescence program in an autocrine manner and to promote senescence induction in a paracrine mode [14,21,135,136,137]. Namely, SASP causes diverse effects in senescent cells and their neighbor cells. Some of the effects are beneficial for tumor suppression, such as the suppression of malignancy in pre-malignant tumor cells, the activation of the immune system to remove damaged cells, and the promotion of wound healing and tissue repair [19,133,138,139,140]. However, detrimental effects, including chronic inflammation, stem cell-like phenotypes in malignant cells, and the promotion of tumor immune evasion and angiogenesis, contribute to tumor promotion [14,21,135,136,137]. These properties are mediated by p53 and nuclear factor-κB (NF-κB) [141]. Zhang and Friedman showed that p53-triggered SASP derived from stromal cells strongly influences epithelial tumorigenesis in the liver [142]. Moreover, Lujambio et al. showed p53 regulates the SASP of hepatic stellate cells that accumulate in the liver and coordinate the production of fibrotic scar tissue, resulting in hepatocellular carcinoma [139]. Thus, the senescence response, particularly SASP, in tumorigenesis is considered a double-edged sword.

Many studies have shown that p53 isoforms are abnormally expressed in breast cancer, ovarian cancer, lung cancer, colon carcinoma, glioblastoma, melanoma, head and neck tumors, renal cell carcinoma, acute myeloid leukemia, and hepatic cholangiocarcinoma [70,84,143,144,145,146,147,148,149,150,151]. These results led us to consider whether each p53 isoform may have different roles in tumorigenesis and cancer through cooperation with full-length p53 or its own direct function. Indeed, Δ40p53 is significantly expressed in the aggressive triple negative (negative expression of estrogen receptor, HER2 (Erb-B2 receptor tyrosine kinese 2), or epidermal growth factor receptor 2, and progesterone receptor) subtype of breast cancer, which is resistant to anti-tumor drugs [152]. Conversely, in wild-type *TP53* mucinous or serous ovarian cancer, higher Δ40p53 expression correlates with better clinical outcomes [153]. Similarly, Δ40p53 expression in melanoma cells and hepatocellular carcinoma cells suppresses their proliferation through the induction of apoptosis or cellular senescence [83,151].

Colon adenomas, which are premalignant tumors associated with senescence, express increased amounts of Δ133p53α compared to normal colon tissues. However, in colon carcinomas, the Δ133p53α expression is comparable with normal colons. This expression change of Δ133p53α is correlated with an expression change of p53β, which is high in colon adenomas and low in colon carcinomas. A further significant increase in Δ133p53α from stage I to II and decrease in p53β from stage II to III carcinomas might have a role in the cancer stage progression. Δ133p53α also stimulates angiogenesis and tumor progression in glioblastoma cell lines and osteosarcoma cell lines, and the expression of angiogenic genes is differentially regulated by the expression ratio of Δ133p53α and p53 [84]. 

The upregulation of Δ133p53α combined with the downregulation of TAp53 (p53α, p53β, and p53γ) is associated with the short patient survival time in cholangiocarcinoma [150]. p53β is correlated with a higher risk of recurrence of wild-type *TP53* ovarian cancer and associated with adverse clinicopathologic markers [148]. In contrast, several studies of different human cancers have shown that prognosis in the *TP53* mutation status is improved with the expression of certain p53 isoforms. The overall survival of mutant *TP53* serous ovarian cancer patients correlates with Δ133p53α expression [154,155]. In breast cancer with mutant *TP53*, higher p53γ expression levels are associated with good prognosis to levels comparable with the wild-type *TP53* status, while the absence of p53γ expression with the mutant *TP53* status is associated with a particularly poor prognosis [149]. Taken together, p53 isoform expression is associated with the clinical outcomes of cancer, which depend on the *TP53* status (wild-type or mutant) and cancer type.

## 6. Concluding Remarks

**Figure 5:**
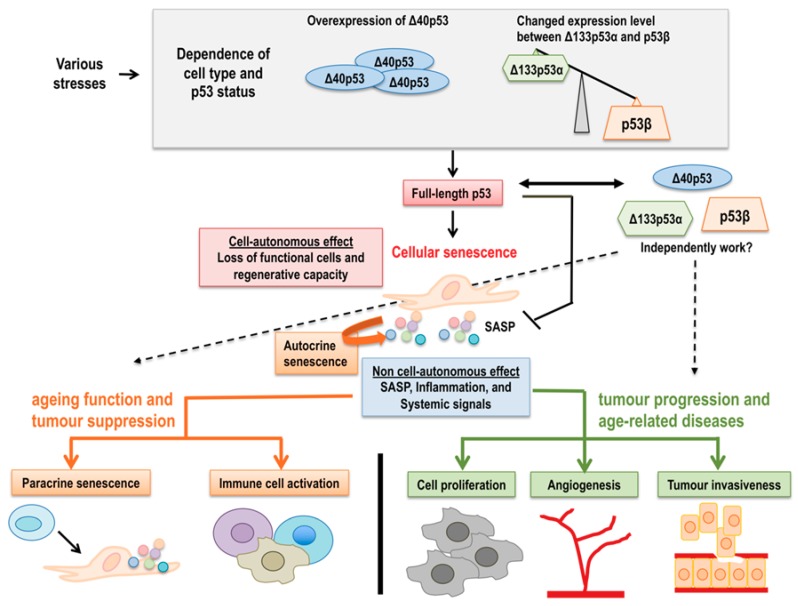
A model for the regulation of cellular senescence, ageing, and age-related disorders by full-length p53 and p53 isoforms. Various stresses induce not only full-length p53 activation, but also changes in p53 isoform expressions depending on the cell type and p53 status, such as abundant Δ40p53 or decreased Δ133p53α and increased p53β, resulting in cellular senescence through cell-autonomous functions including the loss of functional cells and regenerative capacity. Senescent cells also show non cell-autonomous effects, mainly SASP. Autocrine SASP can reinforce senescence, in turn, paracrine SASP influences neighboring cells to induce senescence and activate immune responses, leading to ageing, and tumor suppression. At the same time, SASP also promotes cell proliferation, fibrosis, angiogenesis, and tumor invasiveness, resulting in tumor progression and age-related diseases. This dual effect by cell-autonomous and non-cell-autonomous functions is modulated by full-length p53 and different p53 isoform expressions. Moreover, the different p53 isoform expressions may be crucial for senescence- and age-associated outcomes, and some p53 isoforms may modulate the dual effect of the senescence program dependently or independently of full-length p53.

Cellular senescence is a process in which proliferative-competent cells undergo permanent, irreversible growth arrest in response to stress (for example, replicatively dividing limit, oncogene activation, oxidative stress, or DNA damage) [3,4,5,6]. Senescent cells are distinct from other non-dividing cells by their expression of senescence-associated markers, including short or dysfunctional telomeres, positivity of SA-β-gal, SAHFs, SASP, and activation of the p53 and/or *p16^INK4A^* pathways followed by changed gene expressions [7,8,9,12,13,156,157]. Numerous studies have shown that cellular senescence contributes not only to multiple pathological disorders including cancer, ageing, and age-related diseases, but also to regeneration [4,18,158,159,160,161,162]. In a cell-autonomous manner, senescence acts to deplete various pools of cells in an organism, including stem and progenitor cells, to cause ageing and tumor suppression. Senescence interferes with tissue homeostasis and regeneration, and also in cooperation with non-autonomous factors (i.e., SASP) induces tumor progression and age-related diseases [161]. Emerging evidence has shown that p53 has a key role in the regulation of these cell-autonomous and non-autonomous factors [4,163,164]. p53 modulates cellular senescence at different levels and circumstances with a dual effect, promoting or inhibiting the senescence program. This dual effect seems to depend on the p53 isoform expression pattern. As discussed in this review, some p53 isoforms cooperate with full-length p53, whereas others operate independently. The effect of p53 isoforms on p53-mediated functions against cellular senescence, ageing, and age-related disorders is dependent on the cell type and p53 status. The balance of different p53 isoform expression patterns may be critical for senescence- and ageing-associated outcomes. Moreover, some p53 isoforms modulate full-length p53 transcriptional activity, while others have transcriptional activity independent of full-length p53 even in p53-dependent biological activities (Figure 5). Based on these considerations, there are still many unsolved questions. How are p53 isoforms involved in cancer, ageing, and age-related disorders? How do p53 isoforms and full-length p53-mediated signaling pathways connect with other signaling pathways related to cellular senescence and ageing? Further studies will elucidate the mechanism of p53 isoforms in cellular senescence, ageing, and age-related disorders to enhance our knowledge and advance clinical applications.
